# Modeling of Paper-Based Bi-Material Cantilever Actuator for Microfluidic Biosensors

**DOI:** 10.3390/bios13060580

**Published:** 2023-05-26

**Authors:** Ashutosh Kumar, Hojat Heidari-Bafroui, Nassim Rahmani, Constantine Anagnostopoulos, Mohammad Faghri

**Affiliations:** Microfluidics Laboratory, Department of Mechanical, Industrial and Systems Engineering, University of Rhode Island, 2 East Alumni Avenue, Kingston, RI 02881, USAnara7@uri.edu (N.R.); anagnostopoulos@uri.edu (C.A.)

**Keywords:** paper-based sensor, Bi-Material cantilever, paper-based valve, fluid imbibition, hygroexpansion coefficient, hygroexpansion strain, Whatman Grade 41 filter paper, modulus of paper

## Abstract

This research explores the dynamics of a fluidically loaded Bi-Material cantilever (B-MaC), a critical component of μPADs (microfluidic paper-based analytical devices) used in point-of-care diagnostics. Constructed from Scotch Tape and Whatman Grade 41 filter paper strips, the B-MaC’s behavior under fluid imbibition is examined. A capillary fluid flow model is formulated for the B-MaC, adhering to the Lucas–Washburn (LW) equation, and supported by empirical data. This paper further investigates the stress–strain relationship to estimate the modulus of the B-MaC at various saturation levels and to predict the behavior of the fluidically loaded cantilever. The study shows that the Young’s modulus of Whatman Grade 41 filter paper drastically decreases to approximately 20 MPa (about 7% of its dry-state value) upon full saturation. This significant decrease in flexural rigidity, in conjunction with the hygroexpansive strain and coefficient of hygroexpansion (empirically deduced to be 0.008), is essential in determining the B-MaC’s deflection. The proposed moderate deflection formulation effectively predicts the B-MaC’s behavior under fluidic loading, emphasizing the measurement of maximum (tip) deflection using interfacial boundary conditions for the B-MaC’s wet and dry regions. This knowledge of tip deflection will prove instrumental in optimizing the design parameters of B-MaCs.

## 1. Introduction

Lab-on-a-chip technology, employing innovative materials and components, has made significant strides in diagnosing diseases and detecting a broad range of phenomena [1]. A crucial breakthrough is the development of the Bi-Material cantilever (B-MaC) valve in microfluidic paper-based analytical devices (μPADs), which allows for autonomous control of multiple fluid reagents. This paper-based cantilever, consisting of a sensing and an actuating layer, responds to changes in moisture levels, initiating mechanical motion, a concept inspired by microcantilever sensors in atomic force microscopy [2,3].

B-MaC valves are fundamental to the operation of μPADs for biosensing [4]. Their self-actuation results from hygroexpansion, much like thermal expansion in thermostats. Composite bilayers have found wide-ranging applications in electronics, biomimetics, and biomedical applications [5,6,7,8,9]. Silicon-based μPADs have evolved to develop microactuators and lab-on-a-chip devices [10,11,12,13,14], and various polymers have also been explored [15,16,17,18]. However, nonbiodegradable materials have significant drawbacks, rendering paper-based μPADs attractive due to their biodegradability. Various valving configurations for μPADs have been developed [19,20,21,22,23,24,25]. Paper deformation upon fluid imbibition is a common feature across different applications [26,27,28,29]. Fluid imbibition leads to the expansion of cellulose fibers and subsequent B-MaC bending. This behavior is influenced by solution properties, the paper material, and environmental conditions [30,31], and has been the focus of many studies [32,33,34,35,36,37]. Furthermore, the characteristics of micro check valves have been extensively studied, highlighting their importance in microfluidic systems [38]. The bimaterial cantilever (B-MaC) actuator primarily emphasizes its use in single-use, disposable applications. The design of the paper-based sensor aligns well with low-cost, lightweight, and biodegradable requirements, making it a particularly effective solution in scenarios where the sensor’s reusability might compromise the accuracy of subsequent readings. Such applications could include certain biosensing or environmental monitoring tasks. Furthermore, using the B-MaC design for one-time applications can help avoid potential complications such as contamination, degradation, or material property alterations over multiple uses, which could adversely affect the sensor’s reliability.

This research builds on and extends the recent work on the bending behavior and modeling of these cantilevers [39]. Our research intensively explores the behavior of B-MaCs under fluidic loading, with a specific focus on the impact of material properties, particularly the Young’s modulus. Compared to our previous work, where the wetted length and spatial coordinates were determined assuming the arc length for the radius of curvature of bilayer beam, we now adopt a more nuanced approach. In our current formulation, we utilize the classical beam theory to establish the curvature, factoring in the deflection and curvature of the bilayer beam. We delve deeply into the behavior of B-MaCs under fluidic loading, emphasizing the influence of material properties such as the Young’s modulus. Moreover, we propose a mathematical model that accounts for the hygroexpansive response of the paper [40,41].

One of the significant advancements in our study is the determination of the hygroexpansion coefficient of Whatman 41 paper and the Young’s modulus of both Whatman 41 paper and tape at different moisture levels. These values, previously unreported in the literature, are crucial to accurately understanding the behavior of B-MaCs constructed with these materials. Using arbitrary values does not predict real case deflections accurately; thus, our research fills a critical gap in the existing literature. Despite progress in the field, the current literature lacks a comprehensive model that considers the behavior of bilayers under fluidic loading. Our study introduces a comprehensive model of a fluidically activated B-MaC for the automation of a paper-based assay [42], implemented in a fluidic circuit to sequentially load multiple reagents for analyte detection. By considering various geometric and material properties, our model, which is validated using experimental results, makes a novel and significant contribution to the field. 

## 2. Materials and Methods

A borosilicate capillary is utilized to load a certain amount of fluid onto the paper-based bimaterial cantilever (B-MaC). The sample fluid transfers from the fixed to the free end of the B-MaC due to the capillary action. The fluidic loading of paper-based B-MaC results in the hygroexpansion of cellulose fibers, and the B-MaC starts to deflect over several seconds. In addition, an Instron pull test was performed on a paper-based bimaterial Cantilever (B-MaC) to estimate the Young’s modulus of Whatman Grade 41 filter paper. For this purpose, dog bone samples were utilized with iterations of wet and dry Whatman Grade 41 filter paper and Scotch Tape.

### 2.1. Materials

The following materials were used in preparing, fabricating, and testing the paper-based bimaterial cantilever (B-MaC) valves used in this study: Whatman filter papers grade 41 (GE Healthcare Whatman 41-1441866) purchased from Thermo Fisher Scientific (Waltham, MA, USA); Scotch^®^ Tape 600 (3M, St. Paul, MN, USA); food coloring (Wilton Icing Colors, Illinois, USA) for visual aid; ASTM Type 1 deionized water (resistivity > 18 MΩ/cm, (LabChem-LC267405, Pennsylvania, USA). The dimensions of the cantilevers were cut using Vector 13 graphics software (CorelDraw X6 2022 v24. 1). The cantilevers were then cut out from paper, in a cross-machine direction, using a laser engraver (Epilog mini 40 W 800 Laser System). For material testing, Shimadzu EZ-LX Instron and SCG 1kNA grips were utilized. An 8-megapixel video camera with 30-frames-per-second capability and media player (Avidemux 2.8.1) was used to record and play back the recording and collect the data for the actuation of the cantilever valve.

#### Experiment Flow

**Figure 1:**
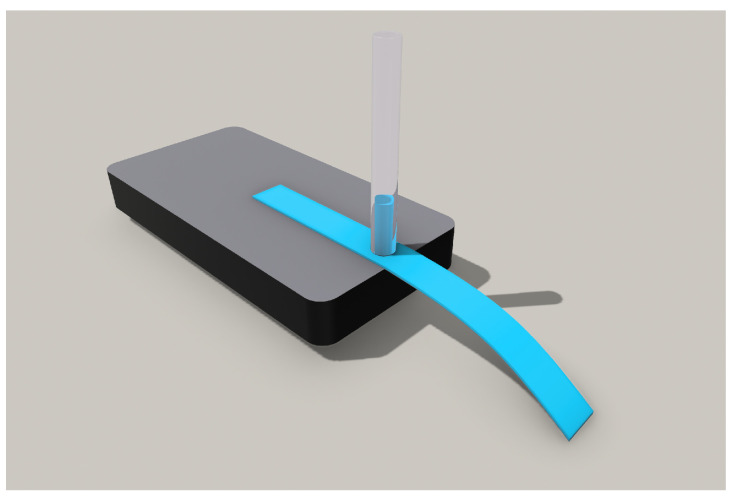
Experimental model representation for paper-based Bi-Material cantilever (B-MaC) valve.

A picture of the experiment model is shown in Figure 1, consisting of a stationary component, paper-based Bi-Material cantilever (B-MaC) valve and capillary tube to load the fluid and obtain the response deflection.

**Figure 2:**
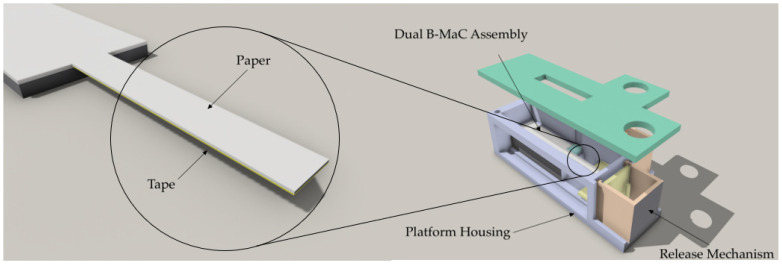
Paper-based Bi-Material cantilever (B-MaC) valve (on **left**); An exploded view of the platform housing [42] (on **right**).

**Figure 3:**
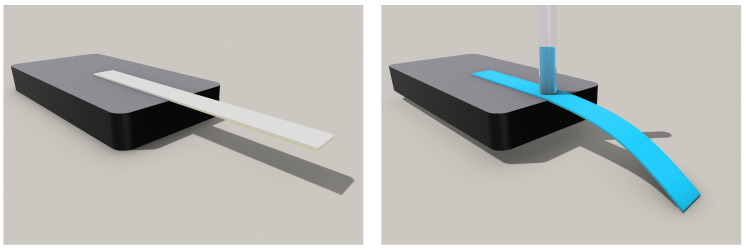
B-MaC before (on **left**) and after (on **right**) fluidic loading.

The Whatman Grade 41 filter paper with one side laminated with tape was cut in a cross-machine direction with a 4 mm width and a 20 mm length using an Epilog Mini laser engraver. The picture of samples for B-MaC can be seen in Figure 2. A 2 mm diameter capillary tube was used to introduce fluid into the paper-based Bi-Material cantilever (B-MaC) valve. The fixture for the positioning of the paper-based cantilever and capillary was designed and utilized to reduce the uncontrollable error of running experiments. Figure 3 shows (on the left) the tape-side-down (normally closed) B-MaC positioned on the fixture in the unloaded condition and (on the right) actuated B-MaC in a loaded condition.

The study was conducted to experimentally assess the Young’s modulus of paper and tape. The modulus for different saturation levels of paper, obtained using six different saturation levels of B-MaC ranging from 0% to 100%, was arbitrarily chosen with steps of 20% increments to cover the possible range of moisture content that a B-MaC can experience in autonomous assays. Moreover, test specimens were loaded onto the Instron machine after a dwell of 30 s to allow the specimens to have even distribution of fluid along the gage dimensions. The paper and tape dog bone test specimens were prepared for the study using Whatman Grade 41 filter paper and Scotch Tape. The filter paper and tape with a 4 mm gage width and a 10 mm gage length were cut using an Epilog Mini laser engraver. A similar setting for moisture content and sample for W-41 filter paper with gauge lengths 10 mm, 20 mm, and 30 mm were utilized to determine the empirical hygroexpansion strain.

### 2.2. Modeling—Bimaterial Cantilever (B-MaC)

For this study, a 2D quasi-static model is adapted with the wetted length as a dependent variable. The experiments were conducted under controlled laboratory conditions, and the effect of temperature and humidity of surroundings are not considered to approach a simpler model. It is important that the model takes dynamic deflection for the B-MaC into consideration; this is handled by using a moving (variable) boundary condition for the wetted length of the cantilever in a 2D model. B-MaC consists of a layer of paper, laminated with tape on one side, and fluidic loading of B-MaC leads to the possibility of delamination. However, significant observation during the experiment assures that the delamination does not occur for the given time for the actuation process; therefore, delamination will not be considered. A classical beam was used to develop the relationship for the curvature of B-MaC, with the assumption that the thickness, h, of B-MaC is small in comparison to the radius of curvature, R; the stress and strain profile in the B-MaC is homogenous; the plane of remains normal before and after bending; and the deflection is the only function of wetted length.

**Figure 4:**
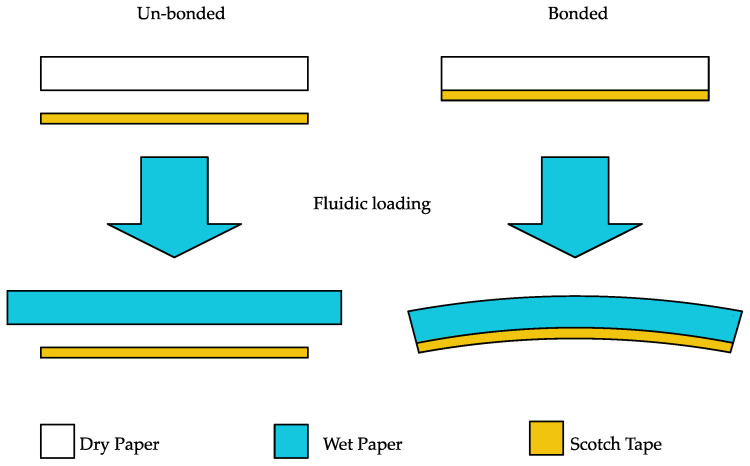
Paper-based bimaterial cantilever (B-MaC) valve upon fluidic loading.

The B-MaC consists of a thin layer of tape (Scotch^®^ Tape 600) laminated on the thinker layer of filter paper (Whatman Grade 41). Figure 4 displays the behavior of the B-MaC upon fluidic loading in two conditions (bonded and unbonded). The B-MaC remains in neutral condition before the fluid is loaded onto the bilayer cantilever. In unbonded conditions the filter paper exhibits hygroexpansion, but no deflection is achieved. However, in bonded conditions, upon loading with fluid, the B-MaC absorbs the sample fluid through wicking and actuates, resulting in bending. The paper layer exhibits hygroexpansion and tape being hydrophobic in nature does not expand. Since the paper and tape layers are bonded together, the hygroexpansion of paper shall be compensated with the inextensible tape layer. Therefore, the generated inconsistency in the strain is responsible for tensile force in the paper layer and compressive strain in the tape layer; these forces and moments generated are equal and opposite in nature to maintain equilibrium.

#### 2.2.1. Strain in B-MaC

Filter paper generally exhibits anisotropic and nonlinear mechanical behavior [43]. The hygroexpansive strain field is locally defined for the filter paper as the relative displacement of the length of the filter paper before and after deformation due to fluid imbibition. On the other hand, the hydrophobic nature of tape does not allow for axial deformation of tape on fluid imbibition.

**Figure 5:**
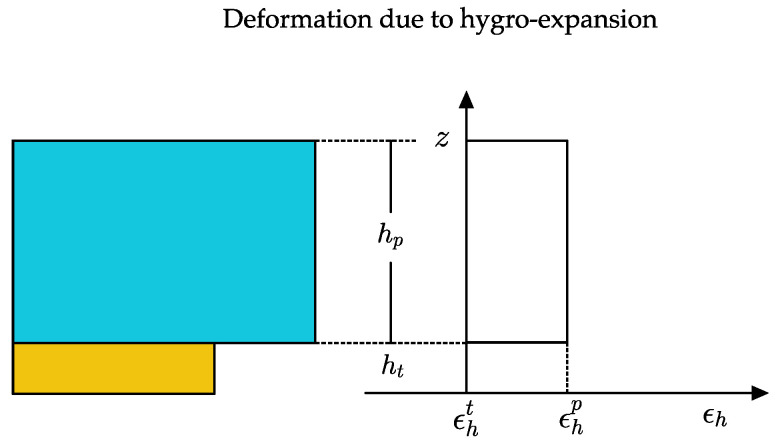
Hygroexpansive strain in B-MaC element.

A schematic representation of the homogenous strain field due to the hygroexpansion of the B-MaC element at 100% saturation is provided in Figure 5. The hygroexpansive strain can be expressed as:

(1) $$\epsilon_{h}=\frac{\Delta l_{h}}{l}$$

where $\Delta l_{h}$ is the change in length due to hygroexpansion and l is the original length. 

Figure 5 represents the hygroexpansion strain in the paper layer and tape layer as $\epsilon_{h}^{p}$ and $\epsilon_{h}^{t}$. Tape being hydrophobic in nature does not exhibit hygroexpansion, resulting in zero value.

**Figure 6:**
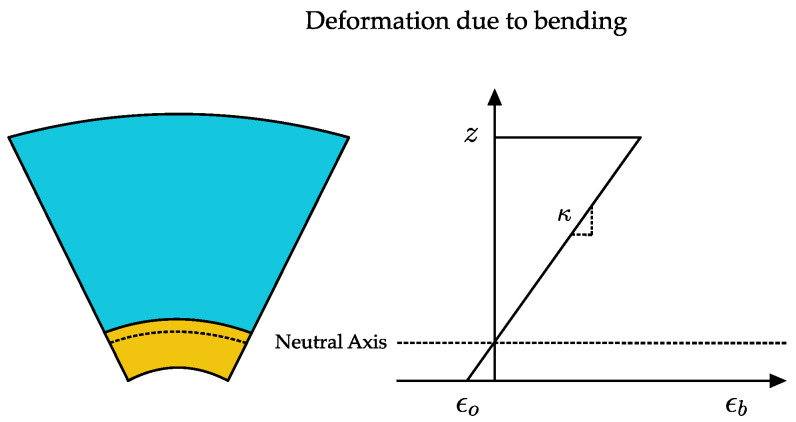
Bending strain in B-MaC element.

The paper and tape layer of B-MaC upon bending restore the bending strain that is responsible for the curvature of B-MaC. In the pure bending state of B-MaC, the bending strain is given in Figure 6:

(2) $$\epsilon_{b}=\epsilon_{o}-z\kappa$$

where $\epsilon_{o}$ is the reference plane strain and κ is the bending curvature.

The total strain in B-MaC under no external load is given by the difference in strain due to bending (Equation (2)) and hygroexpansion (Equation (1)), where positive and negative strain values are the result of tension and compression, respectively.

(3) $$\epsilon =\epsilon_{o}-z\kappa -\epsilon_{h}$$

#### 2.2.2. Stress of B-MaC

The paper upon fluidic loading is subjected to tensile force due to hygroexpansion; the relation for stress and strain for anisotropic paper exhibiting elastic–plastic behavior, established by Ramberg–Osgood form (1943) in slightly modified form, is given by $\epsilon =(\sigma /E)+{\left(\sigma /E_{0}\right)}^{n}$, where E is Young’s modulus, $E_{0}$ is the Hardening modulus, 𝑛 is the Hardening exponent, 𝜎 is the 1D axial stress, and 𝜖 is corresponding strain. Filter paper in elastic range ${\left(\sigma /E_{0}\right)}^{n}$ vanishes from the relation, and future modification of the relation for B-MaC results in

(4) σ=ϵE

#### 2.2.3. Fluid Flow in B-MaC

**Figure 7:**
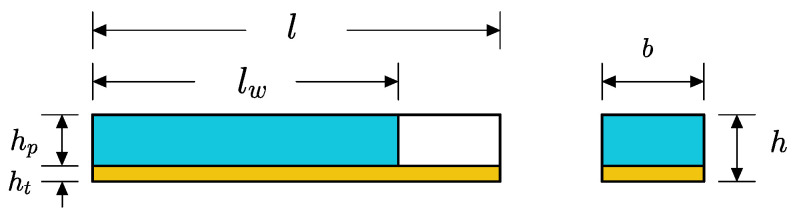
Schematic representation of 2D wetted B-MaC at an arbitrary time.

Fluid imbibition phenomena in paper-like porous material are carried out due to capillary action at the microscale; this is known as pore-level transport. The schematic representation of the samples utilized for the study is provided in Figure 7. Fluid imbibition in B-MaC is an ambiguous phenomenon, and to better understand the fluid flow in B-MaC, we must model the fluid flow for the filter paper layer. The fluid flow in a porous system driven by capillary action is exemplary of fluid flow in filter paper. In this study, the capillary model is adapted for fluid flow into filter paper that obeys the Lucas–Washburn relationship. According to the LW equation [44]:

(5) $$l_{w}=\sqrt{\frac{r\gamma \cos\phi }{2\eta }t}$$

where $l_{w}$—wetted length, *r*—average pore radius, *γ*—surface tension of liquid, *t*—time taken for fluid imbibition, ϕ—contact angle of the liquid on capillary walls and *η*—viscosity of the fluid. The equation can be modified by squaring both sides and using the diffusivity coefficient ψ as $\frac{r\gamma \cos\phi }{2\eta }$.

Simplifying,

(6) $${l_{w}}^{2}=\psi t$$

#### 2.2.4. Material Properties of B-MaC

**Figure 8:**
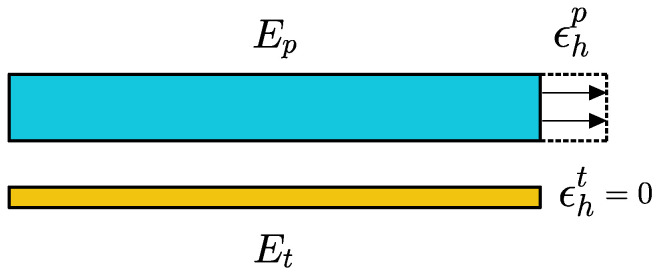
Young’s modulus of fluidically saturated filter paper and tape.

In relation to the material properties of B-MaC, one of the critical aspects is the Young’s modulus of the paper and tape layer of B-MaC. A schematic representation of the fluidically loaded paper and tape layer of B-MaC is provided in Figure 8, where *E_p_* and *E_t_* are the Young’s modulus of fluidically saturated paper and tape respectively. The Young’s modulus is the measure of elastic property defining the ability to withstand the change in length under tension or compressive load before failure. Mathematically, the Young’s modulus of B-MaC can be defined as the ratio of internal axial stress induced to the hygroexpansion strain in the material due to fluidic loading, given by Equation (4).

In conjunction with studying the behavior of B-MaC on fluidic loading, the material properties of paper-based cantilevers were obtained experimentally. For this purpose, an Instron tensile test was performed in laboratory conditions to attain values for the paper-based cantilever’s Young’s modulus.

#### 2.2.5. Linear Coefficient of Hygroexpansion

The tendency of matter to change its shape, area, volume, and density in response to changes in moisture content results in the hygroexpansion of the paper-based cantilever. The ratio of the hygroexpansion strain to the water imbibition content can be defined as the linear coefficient of hygroexpansion. This paper later presents the empirical value of the linear coefficient of hygroexpansion ($\beta_{h}$) for the Whatman Grade 41 paper.

(7) $$\beta_{h}=\frac{\epsilon_{h}}{∆M}$$

where $\beta_{h}$ is the coefficient of linear hygroexpansion and ∆M is the change in moisture content.

### 2.3. Modeling of B-MaC

**Figure 9:**
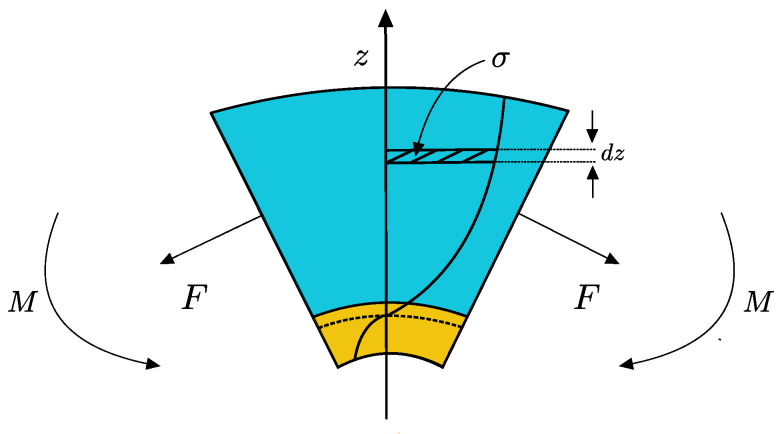
B-MaC element subjected to force and moment due to stress developed in layers.

This paper models the response of B-MaC on fluidic loading of the paper-based cantilever. Our previous work modeled the curvature of the bilayer cantilever utilizing the average intralayer force and moment [39]. This paper presents the model for response deflection considering interfacial conditions of continuous strain and slope between the bilayers and the wet-dry zone, respectively. The wet zone determined by Washburn on fluidic loading models the curvature for coupled fluid and structure bilayer model and utilizes the classical beam relationship to obtain the deflection for a given wetted length. The dry zone is perpetuated as a straight line and utilizes the slope at the interface to obtain the tip deflection of the bilayer cantilever. The modeling of B-MaC determines the response deflection over the period of actuation. Table 1 provides the details of the parameters required for modeling,

Geometry is inspired by the bending of a paper-based cantilever when exposed to fluid [1]. B-MaC actuation is considered as the system output defined by fluid loading, please refer to Table 1 for parameters. A schematic representation of stress, force, and moment in the paper and tape layer that evolved in B-MaC upon fluidic loading is provided in Figure 9.

The force and moment can be obtained by integrating the stress along the cross section of B-MaC.

Force in B-MaC:

(8) $$F=\int_{0}^{h}\sigma \;dz=\int_{0}^{h}E\left(\epsilon_{o}-z\kappa -\epsilon_{h}\right)dz$$

Moment in B-MaC:

(9) $$M=\int_{0}^{h}\sigma \;z\;dz=\int_{0}^{h}E\left(\epsilon_{o}-z\kappa -\epsilon_{h}\right)z\;dz$$

The B-MaC undergoes bending until the hygroexpansion strain attains its maximum value; at equilibrium, the net force and net moment in the cross section of B-MaC is

(10) F=0; ⋯⋯⋯⋯M=0;

on combining Equations (8)–(10) and writing in matrix form,

(11) $$\left[\begin{array}{c} P\;\;-Q\\ Q\;\;-R \end{array}\right]\left[\begin{array}{c} \epsilon_{o}\\ \kappa \end{array}\right]=\left[\begin{array}{c} F_{h}\\ M_{h} \end{array}\right]$$

where

(12) $$P=\int_{0}^{h}E\;dz;\;Q=\int_{0}^{h}E\;z\;dz;\;R=\int_{0}^{h}E\;z^{2}\;dz;$$

(13) $$F_{h}=\int_{0}^{h}E\;\epsilon_{h}\;dz;\;\;M_{h}=\int_{0}^{h}E\;\epsilon_{h}\;z\;dz$$

The reference strain and bending curvature can be obtained by solving Equation (11):

(14) $$\epsilon_{o}=\frac{RF_{h}-QM_{h}}{Q^{2}-PR}$$

(15) $$\kappa =\frac{PM_{h}-QF_{h}}{Q^{2}-PR}$$

To approach a static bilayer bending, Young’s modulus of paper on saturation is considered to be constant. Moreover, the hygroexpansion strain attained at full saturation of paper corresponds to a constant value. The above Equations (8)–(15) were solved using MATLAB, provided as supplementary information. Substituting values from Equations (12) and (13) in Equations (14) and (15), we obtain

(16) $$\epsilon_{o}=\frac{E_{t}\epsilon_{h}^{t}h_{t}\left(E_{t}{h_{t}}^{3}+6E_{p}h_{p}{h_{t}}^{2}+4E_{p}{h_{p}}^{3}+9E_{p}{h_{p}}^{2}h_{t}\right)-E_{p}\epsilon_{h}^{p}h_{p}\left(E_{p}{h_{p}}^{3}-2E_{t}{h_{t}}^{3}-3E_{t}h_{p}{h_{t}}^{2}\right)}{\left({E_{t}}^{2}{h_{t}}^{4}+4E_{t}E_{p}{h_{t}}^{3}h_{p}+6E_{t}E_{p}{h_{t}}^{2}{h_{p}}^{2}+4E_{t}E_{p}h_{t}{h_{p}}^{3}+{E_{p}}^{2}{h_{p}}^{4}\right)}$$

(17) $$\kappa =\frac{{6(E}_{p}E_{t}{)(h}_{p}h_{t})\left(h_{p}+h_{t}\right)(\epsilon_{h}^{t}-\epsilon_{h}^{p})}{\left({E_{t}}^{2}{h_{t}}^{4}+4E_{t}E_{p}{h_{t}}^{3}h_{p}+6E_{t}E_{p}{h_{t}}^{2}{h_{p}}^{2}+4E_{t}E_{p}h_{t}{h_{p}}^{3}+{E_{p}}^{2}{h_{p}}^{4}\right)}$$

where $\left(\epsilon_{h}^{t}-\epsilon_{h}^{p}\right)$ is the actuation strain in B-MaC. Since the tape is hydrophobic in nature, the hygroexpansion strain in tape, $\epsilon_{h}^{t}=0$.

On simplifying,

(18) $$\epsilon_{o}=\left(-\epsilon_{h}^{p}\right)\left[\frac{\left(1-2E_{r}{h_{r}}^{3}-3E_{r}{h_{r}}^{2}\right)}{\left(1+4E_{r}{h_{r}}^{3}+6E_{r}{h_{r}}^{2}+4E_{r}h_{r}+{E_{r}}^{2}{h_{r}}^{4}\right)}\right]$$

(19) $$\kappa =-\left(\frac{\epsilon_{h}^{p}}{h_{p}}\right)\left[\frac{6E_{r}h_{r}(1+h_{r})}{\left(1+4E_{r}{h_{r}}^{3}+6E_{r}{h_{r}}^{2}+4E_{r}h_{r}+{E_{r}}^{2}{h_{r}}^{4}\right)}\right]$$

(20) $$E_{r}=\frac{E_{t}}{E_{p}};\;\;h_{r}=\frac{h_{t}}{h_{p}}$$

(21) $$\left[\frac{6E_{r}h_{r}(1+h_{r})}{\left(1+4E_{r}{h_{r}}^{3}+6E_{r}{h_{r}}^{2}+4E_{r}h_{r}+{E_{r}}^{2}{h_{r}}^{4}\right)}\right]=\kappa_{Bilayer}$$

where $\kappa_{Bilayer}$ is the static curvature of bilayer beam.

**Figure 10:**
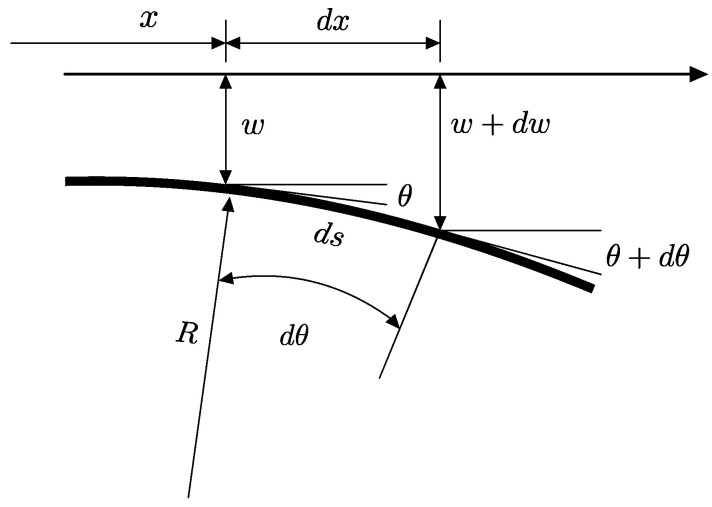
Curvature of B-MaC at any time instance upon fluidic loading.

The bending force and moment contribute to the deflection of B-MaC on fluid imbibition. The placement of tape on B-MaC plays an important role in deciding the curvature of bending; in our case, the bottom surface of filter paper is laminated. A bottom-laminated B-MaC will correspond to negative curvature of bending for B-MaC. From Figure 10, the deflection of B-MaC is given by Equation (A5) (Appendix A):

(22) $$\frac{d^{2}w}{{dx}^{2}}=\kappa$$

Substituting above expression with Equation (19), we obtain

(23) $$\frac{d^{2}w}{{dx}^{2}}=-\left(\frac{\epsilon_{h}^{p}}{h_{p}}\right)\kappa_{Bilayer}$$

Further, the following boundary conditions are accompanied with the governing equations to obtain specific solutions for the modeling of B-MaC. For fixed end (*x* = 0): deflection *w*(*x*) = 0 and slope *w*′(*x*) = 0.

To synthesize the analysis of the modeling, a nondimensionless form is presented in Table 2 below:

Nondimensional governing equations:

Wet zone $\left(0<X<L_{w}\right)$

(24) $$W^{\prime \prime }=-\xi \;\kappa_{Bilayer}$$

where $\xi =\left(\frac{\epsilon_{h}^{p}}{h_{p}}\right)/l$.

Dry zone $\left(L_{w}<X<1\right)$

**Figure 11:**
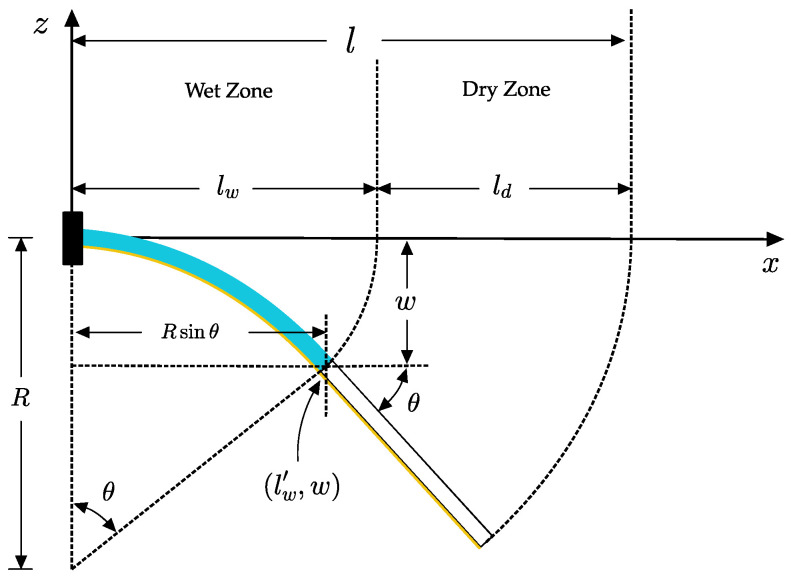
Wet and dry zones of B-MaC.

The dry length is the portion of the B-MaC that the wicking fluid front has not yet reached. Since it is not wetted, it remains straight. As seen in Figure 11, the dry zone length is the difference between the B-MaC total length and the wetted length:

(25) $$\left(y-W\right)=m\left(x-L_{W}^{\prime }\right)$$

where $m={\left(\frac{dw}{dx}\right)}_{{l^{\prime }}_{w}}=W^{\prime }({L^{\prime }}_{w})$.

Boundary conditions:

Fixed-end (x=0):

(26) $$W\left(0\right)=0$$

(27) $$W^{\prime }\left(0\right)=0$$

Interface ($x=L_{w}$):

(28) $${W\left({L_{w}}^{\prime }\right)}_{Wet\;Zone}={W\left({L_{w}}^{\prime }\right)}_{Dry\;Zone}$$

Specific solution for the modeling of B-MaC is evaluated for wet and dry zones using the set of nondimensional equations formulated above with the boundary conditions.

Solution for Equation (24) uses boundary conditions in Equations (26) and (27):

(29) $$W(x)=-\xi \;\kappa_{Bilayer}\frac{x^{2}}{2}$$

To obtain the numerical values for deflection in future, the above solution requires empirical values for modulus for bilayers, hygroexpansion strain, and other geometrical parameters. Details for these parameters are discussed in the following sections.

## 3. Results

This study brings results for two important aspects of B-MaCs, i.e., the material properties and the behavior of the B-MaC on fluidic loading. There are other factors resulting from the experiment conducted for the B-MaC modeling, which will be discussed in detail below. All the results were obtained using the variables in Table 3.

### 3.1. Fluid Flow in B-MaC

**Figure 12:**
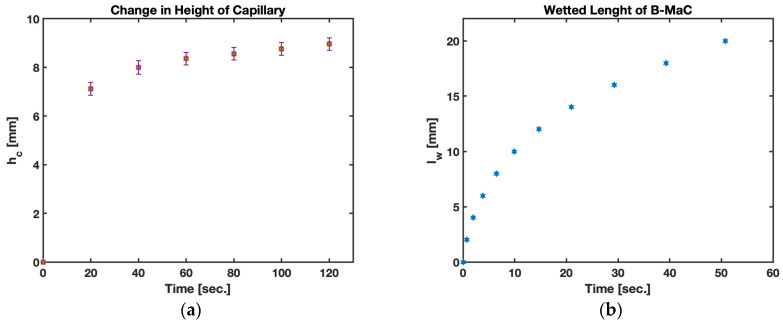
Fluid imbibition in B-MaC (cross-machined direction). (**a**) Change in height of capillary water column *h_c_* (mm) vs. time *t* (sec.); (**b**) wetted length *l_w_* (mm) vs. time *t* (sec.).

The results for fluid flow in the B-MaC that are adapted to the capillary flow model are compared to the experimental results. The two-dimensional rectangular geometry is inspired by the previous cantilever design utilized in μPADs [1]. The rectangular channel model provides good accuracy using the LW theory (Figure 12). The flow is prominently considered only in the lengthwise direction.

**Figure 13:**
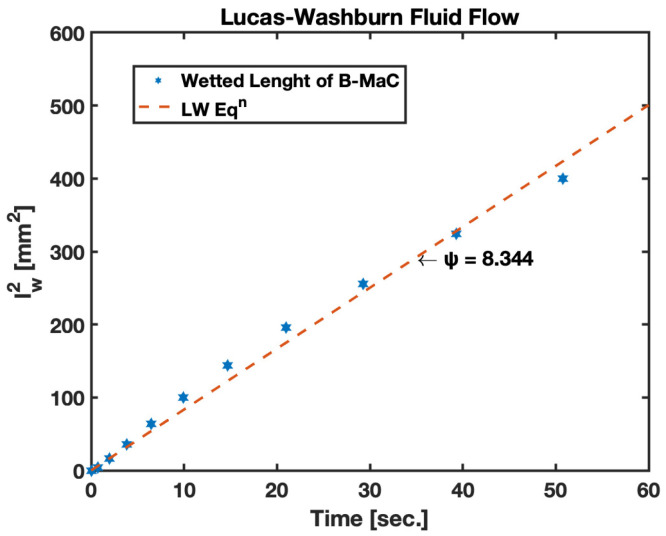
Fluid imbibition in B-MaC (cross-machined direction).

The results for fluid imbibition in the B-MaC (cross machined direction) for the change in height of the capillary water column and wetted length vs. time are presented in Figure 13. In case (a), the change in height of the capillary is inversely proposal to the unsaturated area on the B-MaC; with time, the available unsaturated area of the B-MaC decreases by the relation given in Equation (7). On fluid imbibition, the average change in height of the capillary for the given B-MaC (20 mm × 4 mm) is found to be around 9 mm for 100% saturation. Upon wetting of the B-MaC, the fluid front travels at a rate given by the LW equation. This relation is validated by experimental results obtained for this study, and the plot is presented in Figure 13. The wetted length of the B-MaC is found to be proportional to the sq. root of time, endorsed by the experiment results of this paper. The velocity of fluid imbibition in B-MaC is maximum at the start of fluidic loading and gradually reduced to zero with time, indicating the high volumetric water ingress upon loading B-MaC with fluid at the start and diminishing the volumetric water ingress by the end of loading (or 100% saturation of B-MaC). The experimental results obtained for capillary height and wetted length vs. time are in good agreement with the numerical predictions, shown in Figure 13.

### 3.2. Young’s Modulus of B-MaC

**Figure 14:**
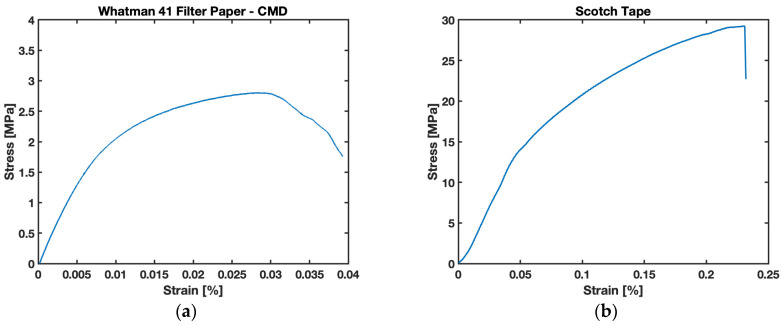
Stress vs. strain relationship: (**a**) Whatman 41 filter paper in CMD; (**b**) Scotch Tape.

The modulus of elasticity is one of the important material properties of B-MaCs that help us predict the behavior of paper-based cantilevers on fluidic loading. Experiments were conducted to obtain stress and strain relationships for the Whatman Grade 41 filter paper in CMD fiber orientation, as well as for Scotch Tape, as shown in Figure 14.

**Figure 15:**
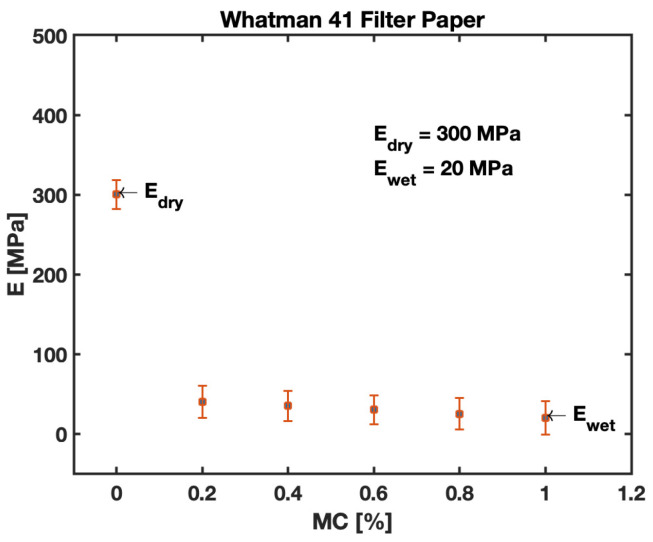
Young’s modulus E (MPa) of Whatman Grade 41 filter paper with different saturation levels MC (%).

The trend illustrated in Figure 14 provides a relation for different zones, i.e., elastic, plastic, stress-hardening, and failure zones. The proportional limit of the stress–strain relationship is utilized to obtain the Young’s modulus of W41 filter paper and Scotch Tape. The experimental values of the Young’s Modulus are found to be ~300 MPa for filter paper in the cross-machine direction and Scotch Tape. Additionally, it is important for the study to obtain the Young’s modulus for saturated filter paper. For this purpose, the study was conducted to acquire values of wetted cantilevers for different saturations (moisture content), and the results are presented in Figure 15. It is evident from the data obtained for the study that the value of the Young’s modulus is drastically affected by the saturation levels of filter paper. The value obtained for the Young’s modulus of saturated filter paper in a cross-machine direction was found to be ~20 MPa. The Young’s modulus drops to ~7% of the respective value for fully (100%) saturated samples. From Figure 15, these drop values of the modulus are very evident for the slight wetting of cantilevers; the instant that fluid is loaded onto the cantilever, the cellulose fibers of the paper start swelling and disintegrating, causing them to lose their rigidity. This justifies the sudden fall in values for the Young’s modulus of the B-MaC and PBC on wetting. These experimental values are utilized for modeling and predicting the behavior of B-MaCs on fluidic loading.

### 3.3. Stress and Strain in B-MaC

The results of this section primarily focus on the behaviors of filter paper on fluidic loading; therefore, further on, we will be discussing results obtained for the important parameters concerning the modeling of B-MaCs. The stress and strain relationships for paper-based cantilevers were discussed in the previous paper [1] and provided a detailed discussion of the considerate factors for modeling PBC on fluidic loading. Unlike PBC, B-MaCs consist of a layer of laminated tape that encourages us to study the effect of a bi-layer cantilever on fluidic loading. Relationships established in the previous sections for effective strain (ϵ) in B-MaCs can be obtained by Equation (3), i.e., the effective strain is the result of the bending and hygroexpansive strain of the B-MaC. The values of bending strain ($\epsilon_{b}$) and hygroexpansion strain ($\epsilon_{h}$) for the B-MaC are carefully deduced empirically and found to be around 0.2% (at tape and paper interface) and 0.8%, respectively.

**Figure 16:**
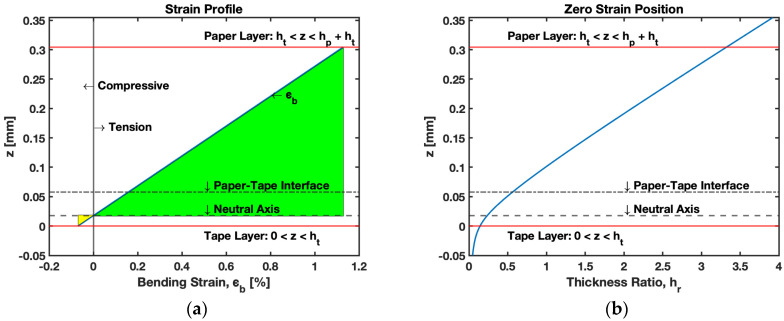
Bending strain in B-MaC. (**a**) Strain profile in thickness of B-MaC; (**b**) zero strain position in B-MaC with varied thickness ratio $h_{r}$.

The strain profile obtained for the wetted cantilever laminated with tape at the bottom is shown in Figure 16a, which provides information on bending strain ($\epsilon_{b}$) in the B-MaC. The figure details that upon fluidic loading of the B-MaC, the paper undergoes hygroexpansion, to which the tape layer’s mechanical response results in a bending state to obtain a sustainable deflection. The green and yellow sections in the plot represent the tension and compression loading respectively in the B-MaC layers. During fluidic loading, the paper layer ($h_{t}<z<h_{p}+h_{t}$) experiences tension loading due to the hygroexpansion of the constituent cellulose fiber of paper, and the tape layer ($0<z<h_{p})$, as depicted, experiences compression loading to compensate for the actuation of the B-MaC. Figure 16b demonstrates the path traced for the zero-strain location in B-MaC for the varied thickness ratio $h_{r}$. The position of zero strains gives us information on the neutral axis. As the $h_{r}$ is varied, the neutral axis moves up from the tape layer to the paper layer.

**Figure 17:**
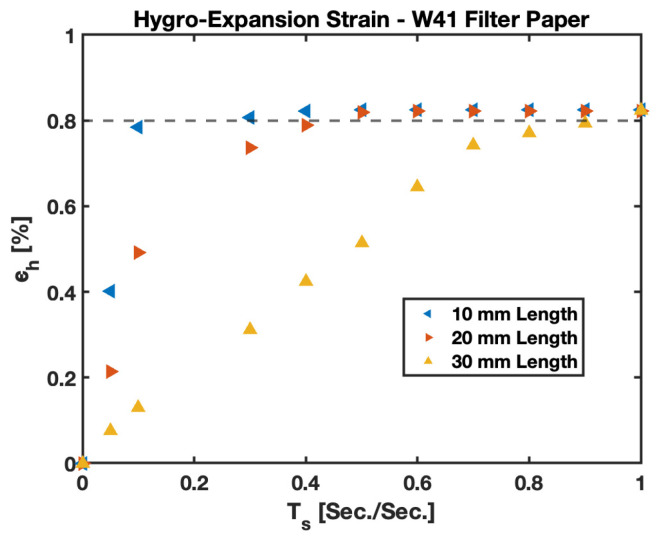
Hygroexpansion strain $\epsilon_{h}$ vs. characteristic time $T_{s}$ for 10 mm, 20 mm, and 30 mm length PBC in Whatman Grade 41 filter paper.

Comparative value plots for the hygroexpansion strain ($\epsilon_{h}$) in W41 filter paper are presented for different lengths in Figure 17. All cases illustrate that irrespective of the length of the cantilever chosen, the hygroexpansion strain ($\epsilon_{h}$) reaches a constant value after a given amount of time. The amount of time required by the cantilever to stabilize $\epsilon_{h}$ values indicate the time taken by the cantilever to fully saturate on fluidic loading. Figure 17 presents the value, $\epsilon_{h}$, for 10 mm, 20 mm, and 3 mm cantilevers. The 10 mm filter paper, being the smallest in length, requires the least amount of time for 100% saturation on fluidic loading, evidently stabilizing the empirical value $\epsilon_{h}$ quickest, whereas 20 mm and 30 mm PBC take more time for saturation on fluidic loading and to reach the steady state value for the hygroexpansion strain ($\epsilon_{h}$). At saturation, the $\epsilon_{h}$ for W41 filter paper is found to be 8×10^−3^±0.5×10^−3^.

**Figure 18:**
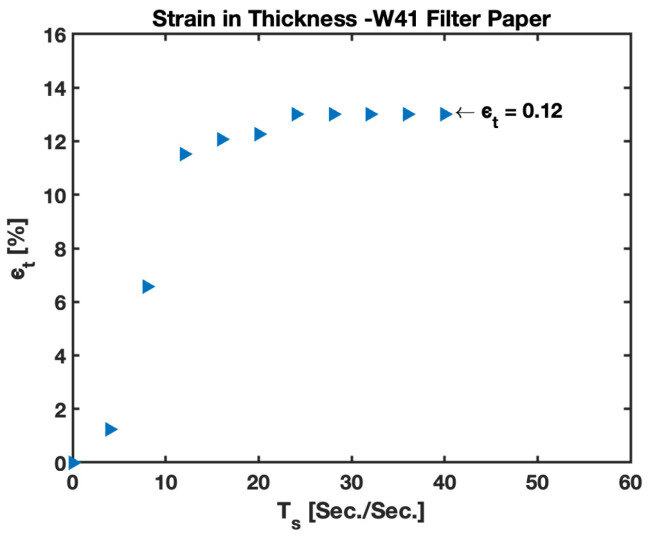
Plot for thickness strain $\epsilon_{t}$ vs. characteristic time $T_{s}$ for Whatman Grade 41 filter paper.

Additionally, it is important to study the strain in thickness for the paper layer of the B-MaC. Upon wetting on the B-MaC, the volumetric change in configuration includes the deformation in thickness, along with the length (considered in previous sections). The strain in thickness ($\epsilon_{t}$) is responsible for choosing the correct second moment of inertia and final thickness of B-MaC modeling. The thickness strain presented in Figure 18 follows a similar trend, with a sudden rise in the value on wetting and reaching a steady-state value of 0.12. As detailed in the previous sections, the fabrication of B-MaCs utilizes W-41; for PBC, the surface of the cantilever is not laminated with tape, encouraging loss of fluid from the surface due to evaporation. However, the B-MaC’s bottom surface is laminated with tape not favoring evaporation from the laminated surface. This experiment is not suitable to capture the loss of source fluid from the specimen; hence, the results will not be discussed in detail due to unsuitable data for the scope of the experiment.

### 3.4. Linear Coefficient of Hygroexpansion Paper

**Figure 19:**
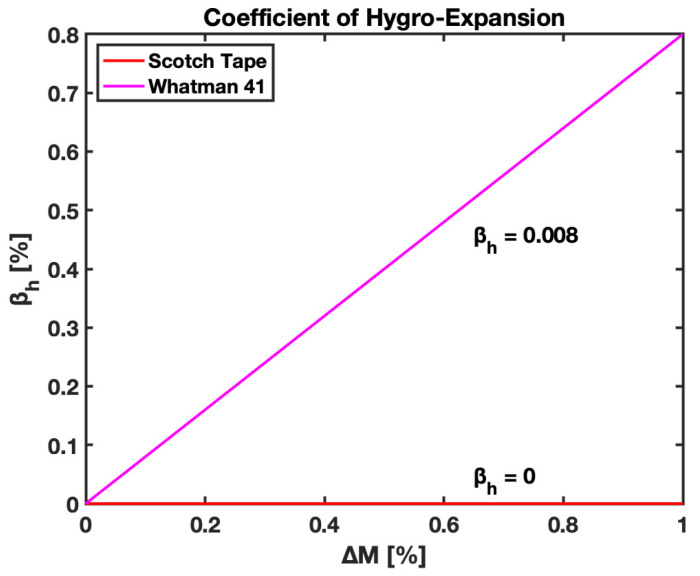
Linear coefficient of hygroexpansion $\beta_{h}$ vs. moisture content ∆M for Whatman Grade 41 filter paper.

The coefficient of hygroexpansion is the ability of paper to change its shape, area, volume, and density in response to moisture change. This definition is tailored to study the change in the length of paper upon wetting, and the linear coefficient of hygroexpansion is introduced, as per Equation (7). This change in the length of the paper is also representative of hygroexpansive strain values at 100% saturation of the cantilever, providing information that can be relayed as the coefficient of hygroexpansion. This study presents the empirical value for the linear coefficient of hygroexpansion ($\beta_{h}$) for Whatman 41 filter paper. The value is found to be 0.008, based on the results obtained for the hygroexpansion strain upon the wetting of the paper. Figure 19 plots the change in the length of the paper to the original length vs. the change in moisture content for the W41 filter paper; empirical data were utilized for the study to obtain the slope of the line expressing the value of $\beta_{h}$ for the W41 filter paper. The coefficient of hygroexpansion helps us understand the mechanical properties of paper used for the fabrication of cantilevers, which further assists in studying the behavior of B-MaC on fluidic loading. 

### 3.5. Deflection of B-MaC

**Figure 20:**
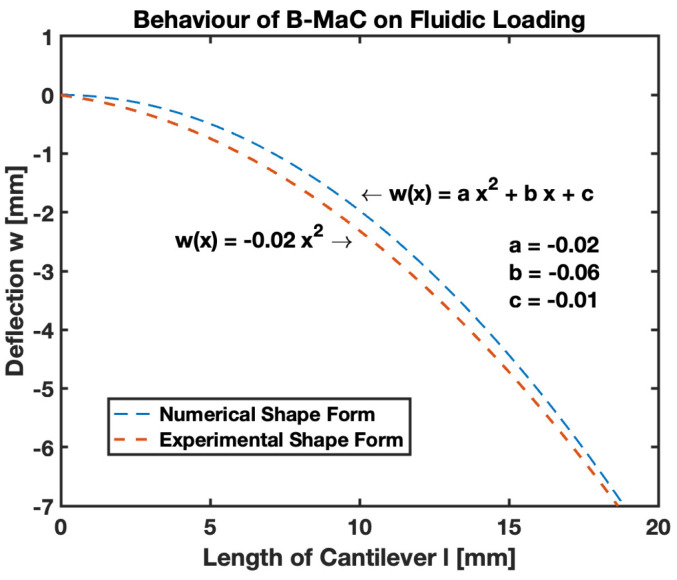
Deflection of B-MaC—numerical and experimental shape forms.

To predict the sustainable deflection of B-MaCs on fluidic loading, theoretic analysis was carried out to obtain results for temporal shape evolutions. These experimental and numerical shape forms presented in Figure 20 illustrate that the observed behavior of B-MaCs can be precisely predicted from the model presented in this paper. The governing nondimensional Equation (25) was utilized to solve the deflection as a function of the wetted length of the cantilever. To obtain response deflection numerically, Neuman boundary conditions for deflection (*w*) and slope (*w*′) of the cantilever at a fixed end were used. However, empirically, the fixed end of the cantilever upon fluidic loading experiences a sight deformation in the geometry of the B-MaC that constrains the use of the Neuman boundary condition. To obtain the best-fitting solution for the accuracy of bending strain, a polynomial fit was utilized in the plot. The result can also be utilized for the modeling of bimaterial cantilevers with the assumption of no stretching in the neutral axis. The modeling of the B-MaC is essential to understand the functionality of microfluidic paper-based analytical devices (μPADs), which are most suited for small–moderate deflection; for this purpose, this paper will focus on results for the deflection of B-MaCs.

**Figure 21:**
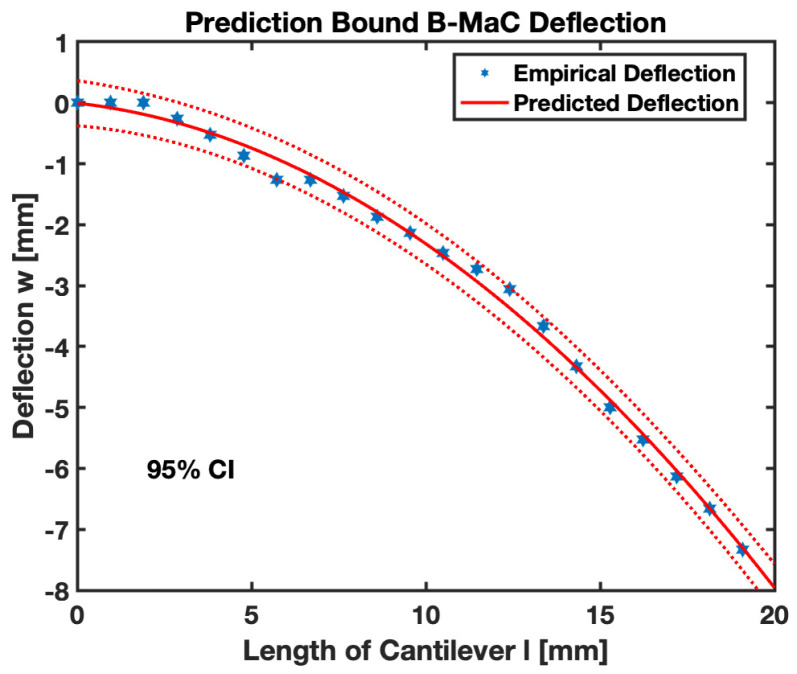
Predicted bound—numerical and experimental deflection of B-MaC.

**Figure 22:**
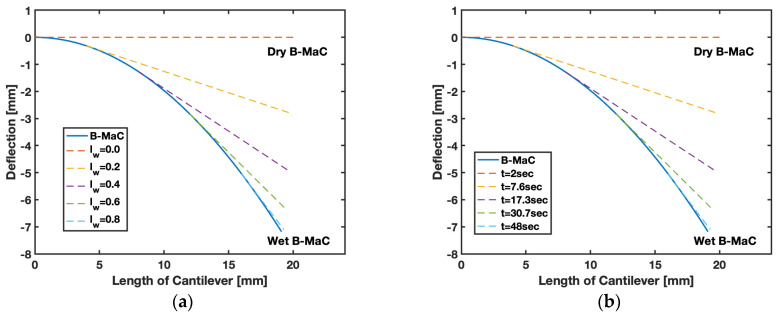
Response deflection of B-MaC. (**a**) For a given wetted length; (**b**) for a given time instance.

In order to validate the obtained results for the response deflection of B-MaCs, data for deflection were obtained experimentally. The numerical values predicted by the model were found to be within the predicted bound, shown in Figure 21. The empirical value for the maximum (tip) deflection of B-MaC is ~7 mm, which is almost three times the value obtained for PBC [1]. Tip deflection is the maximum deflection of the cantilever for any given wetted length. Figure 22a provides tip deflection for 0%, 20%, 40%, 60%, 80%, and 100% wetted lengths $\left(l_{w}\right)$ of B-MaC. Other intermediate cases can be assessed by using Equation (1) for any given time period of fluidic loading, as shown in Figure 22b.

The response deflection of the B-MaC obtained in Equation (29) justifies that the curvature of the cantilever is constant over its length, and the deflection depends on the factors $E_{r}$, $h_{r}$, and ξ. These parameters help in predicting the behavior of B-MaCs on fluidic loading.

### 3.6. Parametric Models

The parametric model helps us understand the material, geometrical, and physical characteristics of B-MaCs under fluidic loading. The modeling of the B-MaC involves three vital parameters: $E_{r}$, $h_{r}$, and ξ, which seek to provide information on the choice of material and dimensional constraints for fabrication and the effect of fluid imbibition. 

**Figure 23:**
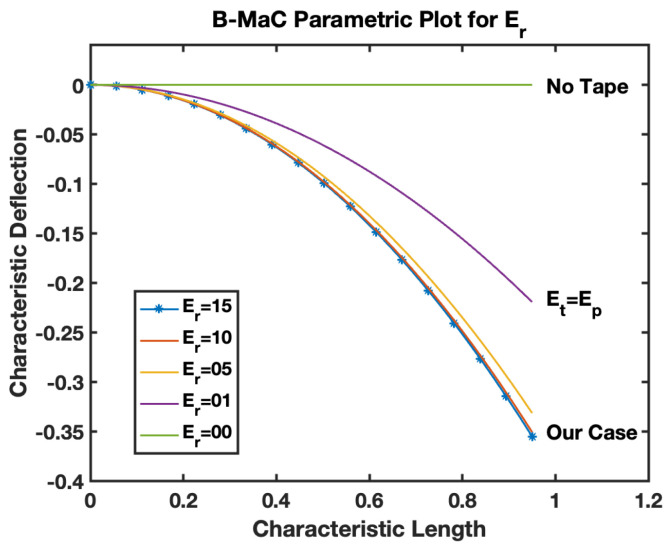
Parametric model for $E_{r}$–B-MaC.

**Figure 24:**
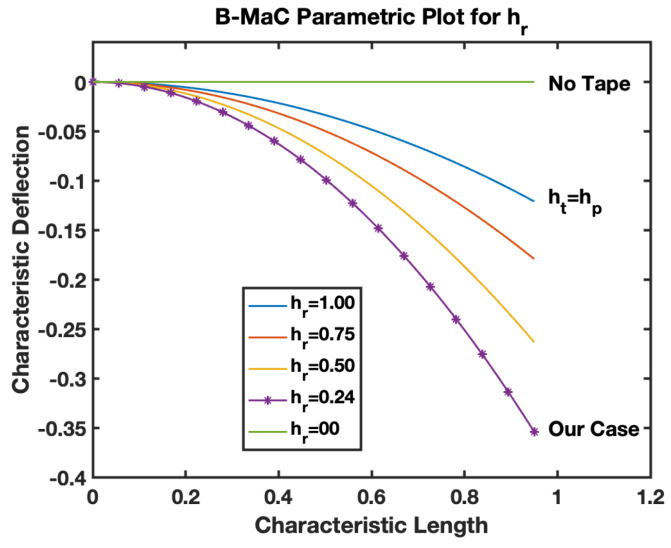
Parametric model for $h_{r}$–B-MaC.

$E_{r}$ is defined by Equation (20), as the ratio of the Young’s modulus of tape to paper used to fabricate the B-MaC. This parameter provides the relative ability of the choice of material to withstand linear deformation of fluid imbibition. Figure 23, the parametric model for $E_{r}$–B-MaC, illustrates the behavior of fluidically loaded B-MaC deflection. The characteristic deflection is plotted against the characteristic length of the B-MaC for different $E_{r}$ ratios of 15, 10, 05, 01, and 0. As the values of $E_{r}$ start dropping, the response deflection of the B-MaC reduces until $E_{r}\to 0$, which is the case of no deflection. $E_{r}\to 0$ signifies a high Young’s modulus of paper with respect to the tape. Cases where the paper is replaced with material of high E value on saturation will result in negligible deflection. $E_{r}=0$ indicates the absence of tape in B-MaC, and the bilayer theory cannot be utilized to obtain a response deflection of B-MaC. Therefore, in such cases, the solution for a small deflection model for a monolayer paper-based cantilever [1] shall be utilized to obtain response deflection. Figure 24 indicates the case utilized for this study, which corresponds with the characteristic deflection of 0.36 for the tip on the B-MaC located at 0.95 characteristic lengths. Additionally, $E_{r}=1$ represents the case for which the modulus of tape and paper are the same, resulting in maximum characteristic deflection ~0.2.

The $h_{r}$ is defined by Equation (20) as the ratio of the thickness of tape to the paper layer of the B-MaC. This parameter provides information on the geometrical parameters utilized for the fabrication of B-MaCs. Figure 24, the parametric model for $h_{r}$–B-MaC, plots the characteristic deflection of the B-MaC on fluidic loading for different $h_{r}$. As shown, for $h_{r}$ from 0 to 1, the maximum characteristic response deflection reduces from 0.36 to 0.

This paper utilizes specific filter paper and tape types, described in the Materials and Methods section. The W-41 paper of thickness 220 μm is laminated by a layer of Scotch Tape of thickness 58 μm; this limits us to ignore the thickness of the tape. However, distinguishing characteristics of these B-MaCs may consist of a thin film layer; for such a case, where $h_{r}<<1$, Equation (24) can be simplified to obtain the deflection for the B-MaC.

**Figure 25:**
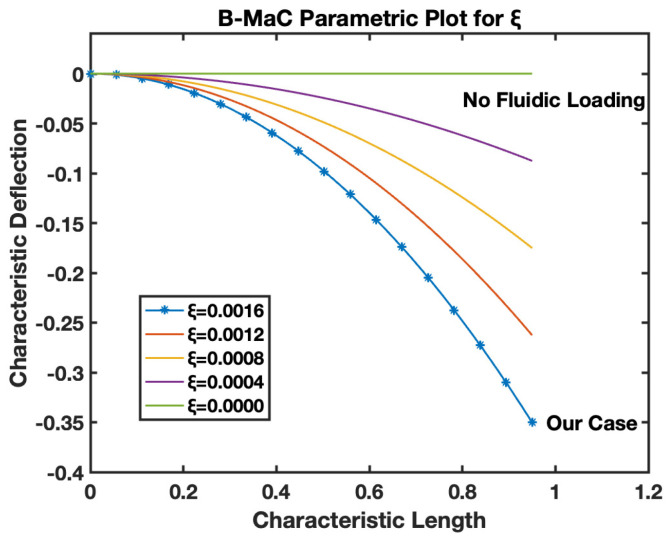
Parametric model for ξ–B-MaC.

The ξ is the ratio of change in length on wetting to the thickness of filter paper used for fabricating the B-MaC. This parameter provides information for the physical characteristics of filter paper. Figure 25, the parametric model for ξ–B-MaC, depicts the response deflection of B-MaC on wetting for different values of ξ. For this study, a ξ value of 0.0016 is obtained for the parameters, which corresponds to a maximum characteristic deflection of 0.36. The plot shows how reducing the value of ξ proportionally decreases the characteristic deflection of the B-MaC. ξ=0, indicating the case of no fluidic loading, which is a result of a null value for the hygroexpansion strain of paper. When the paper is dry before the fluid is loaded onto the paper, the B-MaC remains at rest, corresponding to $\epsilon_{h}^{p}=0$. Upon fluidic loading, the $\epsilon_{h}^{p}$ value increases until reaching a constant value. These increasing values of $\epsilon_{h}^{p}$ will give different ξ; some of these cases are presented in Figure 25.

**Figure 26:**
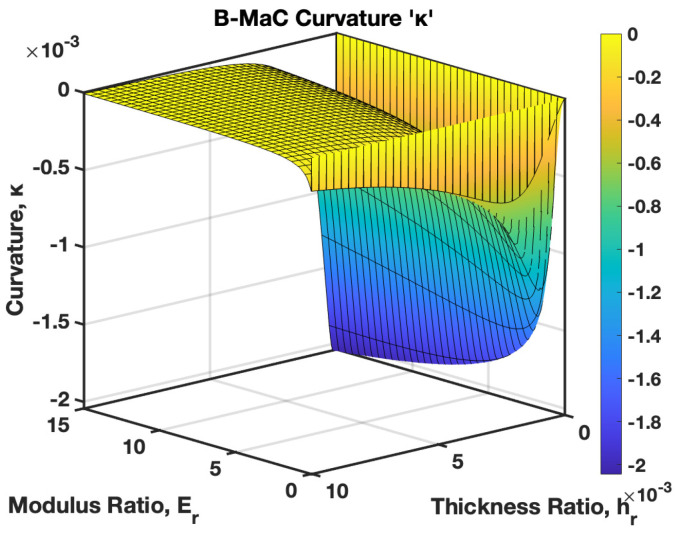
Curvature κ of B-MaC for varied modulus ratio $E_{r}$ (0 to 15) and thickness ratio $h_{r}$ (0 to 10).

It is interesting to notice the effect of the varied modulus ratio $E_{r},$ and the thickness ratio $h_{r}$ together on the curvature of the B-MaC. In Figure 26, the Young’s modulus and thickness ratio are varied from 0–15 to 0–10, respectively. This plot provides important results to select a geometrical parameter for B-MaC fabrication for the purpose of optimizing the curvature of the bilayer beam. A value for $h_{r}\;\mathrm{of}\;0.2$ and $E_{r}\;\mathrm{of}\;15$ will result in the maximum curvature of the B-MaC.

**Figure 27:**
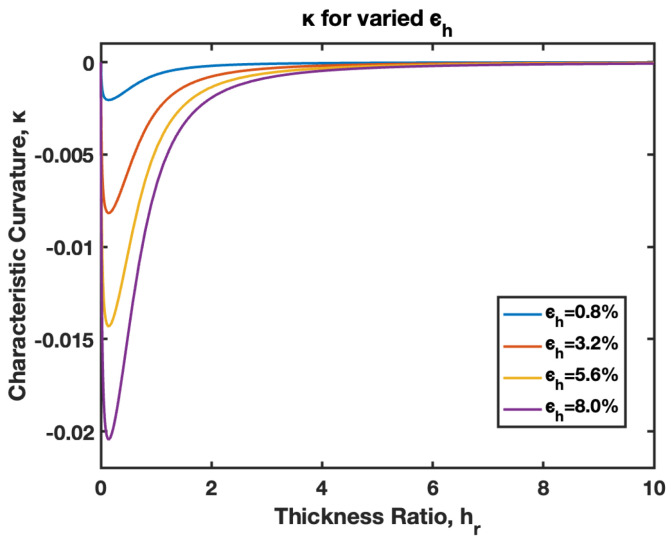
Curvature κ of B-MaC for varied hygroexpansion/actuation strain $\epsilon_{h}$.

The model presented in this paper considers small–moderate deflection of B-MaC, suitable for the application of NO-NC actuator/valves for μPADs and microfluidic assay. This model can be utilized for the large deflection of the cantilever, presented as a limited case for modeling. Figure 27 illustrates the curvature κ of the B-MaC for varied hygroexpansion/actuation strain $\epsilon_{h}$; the curvature obtains its maximum value at $h_{r}\sim 0.2$ for different strains, and decreases in value upon further increasing $h_{r}$. The plot compares curvatures for 0.8%, 3.2%, 5.6%, and 8% of actuation strains. Moreover, the plot demonstrates that increasing the $h_{r}$ beyond the value of 4 hardly affects the curvature of the B-MaC.

## 4. Discussion

This paper predicts the behavior of fluidically loaded B-MaCs and obtains response deflection for the purpose of modeling the moderate deflection of the cantilever. A capillary fluid flow model is considered for B-MaCs obeying the LW equation. Other important material properties, such as Young’s modulus, hygroexpansive strain, and coefficient of hygroexpansion, were assessed to aid the modeling of the B-MaC. The B-MaC moderate deflection model was also assessed with different parameters to provide a full understanding of the change in behavior of response deflection of fluidic loading.

### 4.1. Fluid Flow in B-MaC

It is evident from the results obtained that fluid flow in B-MaC is governed by the LW equation with a good degree of reliability. The change in capillary height helps us understand the volumetric flux of fluid required for the saturation of B-MaC for this study. Upon wetting the filter paper layer of the B-MaC, it indulges in fluid imbibition, and the wetted length of the cantilever is obtained for the loading period. The results for the square of wetted length vs. time show a linear relation with a slope of 0.8344. The empirical data obtained for the plot closely follow this trend, asserting the choice of the capillary flow model for B-MaCs.

### 4.2. Young’s Modulus of B-MaC

The material property helps us understand and predict the response deflection of B-MaCs. We are aware that B-MaCs consist of a layer of filter paper and laminated tape, and understanding the modulus of constituent material provides a complete picture of hygroexpansive strain and stress relationships. The tape layer provides additional rigidity to filter paper, resulting in strengthening the flexural rigidity of B-MaC. The fluidic loading drastically affects the flexural rigidity of paper, and the plots are evident of this reduced value, down to 7%, of dry paper vs. fully saturated paper. The higher values of the modulus for a fully saturated cantilever indicate better flexural rigidity, which leads to reduced response deflection. Similar trends are presented by Lee et al. for Whatman Grade 1 filter paper [45].

### 4.3. Stress and Strain in B-MaC

The stress–strain relationship helps us estimate the modulus of B-MaCs for different saturation levels/moisture content and predict the behavior of a fluidically loaded cantilever. We have learned that two kinds of strains, i.e., hygroexpansion and bending, are involved in a fluidically loaded B-MaC. The hygroexpansion strain helps us understand the change in length of paper upon wetting, and the bending strain provides details regarding the shape form of the cantilever. The hygroexpansion of W41 filter paper for different lengths of cantilever shows a similar response over time. The values of hygroexpansive strain increase until a stagnant value is achieved. For W41 filter paper, a value of 0.008 for hygroexpansive strain is attained. The bending strain is evident in the presence of laminated tape in the B-MaC. The bending strain results in 0 when no tape is present, or the hygroexpansive strain predominantly results in an effective strain in the absence of a tape layer. Lee et al. reports similar trends for Whatman Grade 1 filter paper.

### 4.4. Linear Coefficient of Hygroexpansion

As stated in this paper, the linear coefficient of hygroexpansion is the tendency of paper to change length upon wetting. The B-MaC is fabricated using W41 filter paper and a layer of Scotch Tape, and the linear coefficient of hygroexpansion is obtained for paper and tape upon wetting to understand the relative trends. $\beta_{h}=0$ for tape shows its hydrophobic nature; therefore, it does not involve hygroexpansion. Comparatively, W41 filter paper has $\beta_{h}=0.008$, reflecting the hydrophilic nature of paper. 

### 4.5. Deflection of B-MaC

This paper brings important results in terms of the moderate response deflection of B-MaCs on fluidic loading. The experimental results obtained for the deflection of B-MaCs validate the predicted response of the B-MaC’s mathematical model. The 2D quasi-static model provides a specific solution for the response deflection of the cantilever as a function of its characteristic length for given free fixed-end boundary conditions. The numerical and experimental shape forms were compared to understand the crucial details of inconsistent boundary conditions. The polynomial solutions for the response deflection assert the absence of the Neumann boundary condition of a slope at the fixed end; the mathematical model obtains the solution for the deflection with the boundary conditions of zero slopes and deflection at the fixed end. The filter paper, being docile on wetting, tends to soften and lose its rigidity, leading to a slight variation (nonzero boundary condition) at the fixed end of the cantilever. The moderate deflection model presented in this paper predicts the behavior of the B-MaC upon fluidic loading with a high degree of confidence. The empirical values obtained for the deflection fall under the 95% prediction bound, validating the adapted model for the B-MaC’s deflection. The maximum (tip) deflection of the cantilever is a critical parameter that is obtained by using the interfacial boundary conditions for the wet and dry regions of B-MaC. The dry length of the cantilever remains unaffected by the fluidic loading and behaves as a straight line with rotation. The rotation is defined by the slope of the wetted length of the cantilever. The results for the tip deflection of dry length, corresponding to 0% to 100%, with an increment of 20% wetted length of the B-MaC, are presented to predict the behavior of the B-MaC upon wetting. Tip deflection is crucial to determine the design parameters for μPAD’s assays. The B-MaCs are utilized as NO/NC valve actuators in microfluidic assays [3] for the sequential loading of reagents onto sample pads and detection zone. Along with the tip deflection, the rate of loading and unloading of reagents onto the reagent pads via B-MaCs is crucial to determine. The fluid flow models presented in this paper predict this with the wetted length on the cantilever, which is responsible for the loading time and the time to attain the response deflection for a given B-MaC. 

### 4.6. Parametric Models

The behavior of the B-MaC is mainly asserted for three parameters in this paper, namely the material properties, geometrical properties, and physical properties of fabricated materials utilized for the cantilever. The material properties of B-MaCs in both layers help us predict the response of the cantilever, and the relative modulus value of the cantilever provides an understanding of their relative stiffnesses. The $E_{r}$ as defined is the ratio of the Young’s modulus of tape and paper, and B-MaC consists of a layer of paper laminated with tape; therefore, practically, $E_{r}=1$ can be achieved when the values of the modulus of saturated paper will same as the tape modulus. In cases where $E_{p}>>E_{t}$, $E_{r}\to 0$, which will result in no response deflection of the B-MaC. Along with material properties, geometrical parameters are an important factor in accessing the response deflection of the B-MaC. $h_{r}$ is given by the ratio of the thickness of paper to tape of the B-MaC and provides the relative sizes of the cantilever that come together in the formation of the B-MaC. The value $h_{r}$= 1 indicates identical paper and tape layer thickness, which results in ~0.1 maximum characteristic deflection. A thin film approximation is provided for applications where the substrate layer thickness is negligible; the simplified model for thin film approximation can be utilized to obtain the response deflection. Lastly the ξ parameter provides the physical aspect to understand the hygroexpansion of paper upon wetting to the thickness of paper utilized for the fabrication of B-MaCs. B-MaCs with papers with high expansion on fluidic loading and the tape layer will result in a higher response deflection; this result can be inferred from the increasing values of ξ. This study depicts the response deflection of the cantilever, with ξ varying from 0 to 0.0016. Ideally, a high B-MaC hygroexpansion will result in greater response deflection if the thickness of the paper layer is kept constant. This statement can be validated by comparing the deflection of the B-MaC in the machine and cross-machine direction [3].

### 4.7. Limitation of Model

In our study, the modeling was primarily focused on water-based solutions given their ubiquity in microfluidic biosensors. We did not consider other liquids such as oils due to their substantially different physical properties, including viscosity and surface tension, which would require considerable modifications to our current model to accurately depict their interaction with the Bi-Material cantilever (B-MaC). However, we acknowledge the potential of other fluids and foresee the expansion of our model to include such liquids in future work to broaden its applicability across various applications.

We opted for analytical modeling to comprehend the fundamental principles governing the behavior of Bi-Material cantilevers (B-MaCs) under fluidic loading. While this approach affords broad applicability and computational efficiency, we recognize that it might not capture all complexities and subtle effects inherent in bilayer systems, which may be better addressed through simulation software. Furthermore, the current scope of simulation tools for bilayer cantilevers remains somewhat limited. As such, future studies could substantially benefit from incorporating simulation methodologies. Building upon the groundwork of our current bilayer cantilever model under fluidic loading, simulation could serve as a logical next step for in-depth analysis, providing further validation and potentially extending the applicability of our research.

## 5. Conclusions

This research developed a comprehensive model to predict the behavior of a Bi-Material cantilever (B-MaC) under fluidic loading. The B-MaC, fabricated using Whatman 41 (W41) filter paper and Scotch Tape, demonstrated intriguing properties under fluidic loading. The interplay between these materials and fluidic loading became central to the understanding of the B-MaC’s behavior. We significantly advanced our understanding of B-MaC behavior, as our model provided a specific solution for the response deflection of the cantilever. This was confirmed by experimental findings, with empirical values falling within the 95% prediction bound, asserting the credibility of our mathematical model. Furthermore, our investigation unveiled critical insights regarding the material properties of the constituent elements of B-MaCs. Specifically, the Young’s modulus of W41 filter paper was found to decrease to approximately 20 MPa, which is about 7% of its dry state value, upon full saturation. This drastic reduction in flexural rigidity due to fluidic loading, along with the hygroexpansive behavior of W41 filter paper (which we empirically deduced to be 0.008), played a decisive role in determining the deflection of the B-MaC. We also examined different lengths of bilayer cantilever for strains in B-MaCs, including hygroexpansion and bending strains. These investigations were crucial for predicting the B-MaC’s behavior under different saturation levels/moisture contents. Moreover, we conducted an in-depth analysis of the parameters affecting the response deflection. By altering these parameters, such as the ratio of the Young’s modulus of tape to the paper layer ($E_{r}$) and the ratio of tape to the paper layer height ($h_{r}$), we can optimize the curvature of B-MaCs for specific applications. This study’s significance is multifold. It opens new possibilities for the development of compact, low-cost, and biodegradable microfluidic devices. Our ability to predict and manipulate B-MaC behavior allows for the design and optimization of these devices for various applications, such as environmental monitoring and point-of-care diagnostics.

For future research, we propose investigating alternative materials for B-MaCs, improving the model, and considering more complex interactions. These steps could lead to a more comprehensive understanding of B-MaC behavior under different conditions and further enhance microfluidic device versatility and effectiveness. In conclusion, our work has elucidated the potential of B-MaCs for microfluidic biosensors. The findings of this study, coupled with our developed model, are poised to pave the way for future advancements in the field of microfluidics.

## Figures and Tables

**Table 1:** B-MaC parameters.

Variables	Description	Dimension
*l*	Length of B-MaC	L
*w*	Deflection in y	L
*u*	Deformation in x	L
*R*	Bending radius	L
*l_w_*	Wetted length	L
*b*	B-MaC width	L
*h_t_*	Tape layer thickness	L
*h_p_*	Paper layer thickness	L
*t_s_*	Time required for saturation	T
σ	Stress per unit length	ML^−2^T^−2^
*F_h_*	Hygroexpansion force	MLT^−2^
*M_h_*	Bending moment due to actuation	ML^2^T^−2^
*E_t_*	Young’s modulus of tape	ML^−1^T^−2^
*E_p_*	Young’s modulus of saturated paper	ML^−1^T^−2^

**Table 2:** B-MaC nondimensional parameters.

Variables	Expression	Description
*L_w_*	$\frac{l_{w}}{l}$	Characteristic Wetted Length
*W*	$\frac{w}{l}$	Characteristic Deflection
*H*	$\frac{h}{l}$	Characteristic Thickness
*T*	$\frac{t}{t_{s}}$	Characteristic Time

**Table 3:** Variables utilized for the study.

Variables	Description	Value
l	Length of B-MaC	20 mm
*b*	B-MaC width	4 mm
$h_{t}$	Tape layer height	$58\times 10^{-3}\;\mathrm{mm}$
$h_{p}$	Paper layer wetted height	$246\times 10^{-3}\;\mathrm{mm}$
$E_{t}$	Young’s modulus of tape	300 MPa
$E_{p}$	Young’s modulus of saturated paper	20 MPa
$\epsilon_{h}^{p}$	Hygroexpansion strain in paper	0.008

## Data Availability

Data are contained within the article. Additional data not presented in this article are available on request from the corresponding author.

## Appendix A

From Figure 10:

(A1)
ds=R·dθ

Radius of curvature:

(A2)
$$\kappa =\frac{1}{R}=\frac{d\theta }{ds}$$

(A3)
$$ds=\frac{dx}{\cos\theta }$$

Substituting Equation (A3) in (A2), we obtain

(A4)
$$\kappa =\frac{1}{R}=\frac{d\theta }{dz}\cos\theta$$

For small θ, cos⁡θ≈1:

(A5)
$$\kappa =\frac{d\theta }{dx}=\frac{d\left(\frac{dw}{dx}\right)}{dx}=\frac{d^{2}w}{{dx}^{2}}$$

## References

[1] Kumar A., Heidari-Bafroui H., Charbaji A., Rahmani N., Anagnostopoulos C., Faghri M. (2021). Numerical and Experimental Modeling of Paper-Based Actuators. Chem. Proc..

[2] Binnig G., Quate C.F., Gerber C. (1986). Atomic force Microscope. Phys. Rev. Lett..

[3] Heidari-Bafroui H., Kumar A., Charbaji A., Smith W., Rahmani N., Anagnostopoulos C., Faghri M. (2022). A Parametric Study on a Paper-Based Bi-Material Cantilever Valve. Micromachines.

[4] Calleja M., Kosaka P.M., Paulo A.S., Tamayo J. (2012). Challenges for nanomechanical sensors in biological detection. Nanoscale.

[5] Xu R., Lee J.W., Pan T., Ma S., Wang J., Han J.H., Ma Y., Rogers J.A., Huang Y. (2017). Designing Thin, Ultrastretchable Electronics with Stacked Circuits and Elastomeric Encapsulation Materials. Adv. Funct. Mater..

[6] Quirion D., Manna M., Hidalgo S., Pellegrini G. (2020). Manufacturability and Stress Issues in 3D Silicon Detector Technology at IMB-CNM. Micromachines.

[7] Zhang L., Pan J., Liu Y., Xu Y., Zhang A. (2020). NIR–UV Responsive Actuator with Graphene Oxide/Microchannel-Induced Liquid Crystal Bilayer Structure for Biomimetic Devices. ACS Appl. Mater. Interfaces.

[8] Wang L., Wang D., Huang S., Guo X., Wan G., Fan J., Chen Z. (2019). Controllable Shape Changing and Tristability of Bilayer Composite. ACS Appl. Mater. Interfaces.

[9] Morteza A. (2019). Functional Nanomaterial Composites for Soft Sensing and Actuation. Ph.D. Thesis.

[10] Liu F., Alici G., Zhang B., Beirne S., Li W. (2015). Fabrication and characterization of a magnetic micro-actuator based on deformable Fe-doped PDMS artificial cilium using 3D printing. Smart Mater. Struct..

[11] Makino E., Mineta T., Mitsunaga T., Kawashima T., Shibata T. (2011). Sphincter actuator fabricated with PDMS/SMA bimorph cantilevers. Microelectron. Eng..

[12] Maleki T., Chitnis G., Ziaie B. (2011). A batch-fabricated laser-micromachined PDMS actuator with stamped carbon grease electrodes. J. Micromech. Microeng..

[13] Bhattacharjee N., Urrios A., Kanga S., Folch A. (2016). The upcoming 3D- printing revolution in microfluidics. Lab Chip.

[14] Mirzaee I., Song M., Charmchi M., Sun H. (2016). A microfluidics-based on-chip impinger for airborne particle collection. Lab Chip.

[15] Pandya H.J., Park K., Desai J.P. (2015). Design, and fabrication of a flexible MEMS-based electro-mechanical sensor array for breast cancer diagnosis. J. Micromech. Microeng..

[16] Gaitas A., Malhotra R., Pienta K. (2013). A method to measure cellular adhesion utilizing a polymer micro-cantilever. Appl. Phys. Lett..

[17] Kim J.Y.H., Nandra M., Tai Y.C. IEEE, Cantilever Actuated by Piezoelectric Parylene-C. Proceedings of the 25th IEEE International Conference on Micro Electromechanical Systems (MEMS).

[18] Shang Y.F., Ye X.Y., Feng J.Y., Zhou H.Y., Wang Y. (2014). Fabrication and Characterization of a Polymer/Metal Bimorph Microcantilever for Ultrasensitive Thermal Sensing. IEEE Sens. J..

[19] Li X., Tian J.F., Nguyen T., Shen W. (2008). Paper-Based Microfluidic Devices by Plasma Treatment. Anal. Chem..

[20] Jahanshahi-Anbuhi S., Chavan P., Sicard C., Leung V., Hossain S.M.Z., Pelton R., Brennan J.D., Filipe C.D. (2012). Creating fast flow channels in paper fluidic devices to control the timing of sequential reactions. Lab Chip.

[21] Han K.N., Choi J.-S., Kwon J. (2016). Three-dimensional paper-based slip device for one-step point-of-care testing. Sci. Rep..

[22] Martinez A.W., Phillips S.T., Nie Z., Cheng C.-M., Carrilho E., Wiley B., Whitesides G.M. (2010). Programmable diagnostic devices made from paper and tape. Lab Chip.

[23] Rodriguez N.M., Wong W.S., Liu L., Dewar R., Klapperich C.M. (2016). A fully integrated paper fluidic molecular diagnostic chip for the extraction, amplification, and detection of nucleic acids from clinical samples. Lab Chip.

[24] Jayawardane B.M., Wei S., McKelvie I.D., Kolev S. (2014). Microfluidic Paper-Based Analytical Device for the Determination of Nitrite and Nitrate. Anal. Chem..

[25] Fu H., Song P., Wu Q., Zhao C., Pan P., Li X., Li-Jessen N.Y.K., Liu X. (2019). A paper-based microfluidic platform with shape-memory-polymer-actuated fluid valves for automated multi-step immunoassays. Microsyst. Nanoeng..

[26] Bowden N., Brittain S., Evans A.G., Hutchinson J.W., Whitesides G.M. (1998). Spontaneous formation of ordered structures in thin films of metals supported on an elastomeric polymer. Nature.

[27] Efimenko K., Rackaitis M., Manias E., Vaziri A., Mahadevan L., Genzer J. (2005). Nested self-similar wrinkling patterns in skins. Nat. Mater..

[28] Huang J., Juszkiewicz M., de Jeu W.H., Cerda E., Emrick T., Menon N., Russell T.P. (2007). Capillary wrinkling of floating thin polymer films. Science.

[29] Holmes D.P., Roche M., Sinha T., Stone H.A. (2011). Bending and twisting of soft materials by non-homogenous swelling. Soft Matter.

[30] Siddique J.I., Anderson D.M., Bondarev A. (2009). Capillary rise of a liquid into a deformable porous material. Phys. Fluids.

[31] Ma J., Zhang C., Xi F., Chen W., Jiao K., Du Q., Bai F., Liu Z. (2022). Experimental Study on the Influence of Environment Conditions on the Performance of Paper-Based Microfluidic Fuel Cell. SSRN Electron. J..

[32] Liu Z., Hu J., Zhao Y., Qu Z., Xu F. (2015). Experimental and numerical studies on liquid wicking into filter papers for paper-based diagnostics. Appl. Therm. Eng..

[33] Cheng H., Liu J., Zhao Y., Hu C., Zhang Z., Chen N., Jiang L., Qu L. (2013). Graphene Fibers with Predetermined Deformation as Moisture-Triggered Actuators and Robots. Angew. Chem. Int. Ed..

[34] Leppa T., Sorvari J., Erkkila A., Ha J. (2005). Mathematical modeling of moisture induced out-of-plane deformation of a paper sheet. Model. Simul. Mater. Sci. Eng..

[35] Madsen B., Hoffmeyer P., Lilholt H. (2012). Hemp yarn reinforced composites-III. Moisture content and dimensional changes. Compos. Part A-Appl. Sci. Manuf..

[36] Yao Y., Chen X., Guo H., Wu Z., Li X. (2012). Humidity sensing behaviors of graphene oxide-silicon bi-layer flexible structure. Sens. Actuators B Chem..

[37] Duigou L., Castro M. (2015). Moisture-induced self-shaping flax-reinforced polypropylene biocomposite actuator. Ind. Crops Prod..

[38] Mao Z., Yoshida K., Kim J.W. (2019). A micro vertically-allocated SU-8 check valve and its characteristics. Microsyst. Technol..

[39] Chen G., Kumar A., Heidari-Bafroui H., Smith W., Charbaji A., Rahmani N., Anagnostopoulos C., Faghri M. (2023). Paper-Based Bi-Material Cantilever Actuator Bending Behavior and Modeling. Micromachines.

[40] Gendron G., Dano M.L., Cloutier A. (2004). A numerical study of the hygro- mechanical deformation of two cardboard layups. Compos. Sci. Technol..

[41] Dano M.-L., Bourque J.-P. (2009). Deformation behavior of paper and board subjected to moisture diffusion. Int. J. Solids Struct..

[42] Heidari-Bafroui H., Kumar A., Hahn C., Scholz N., Charbaji A., Rahmani N., Anagnostopoulos C., Faghri M. (2023). Development of a New Lab-on-Paper Microfluidics Platform Using Bi-Material Cantilever Actuators for ELISA on Paper. Biosensors.

[43] Mäkelä P., Östlund S. (2003). Orthotropic elastic-plastic material model for paper materials. Int. J. Solids Struct..

[44] Washburn E.W. (1921). The dynamics of capillary flow. Phys. Rev..

[45] Lee M., Kim S., Kim H.-Y., Mahadevan L. (2016). Bending and buckling of wet paper. Phys. Fluids.

